# A role for Ras in inhibiting circular foraging behavior as revealed by a new method for time and cell-specific RNAi

**DOI:** 10.1186/s12915-015-0114-8

**Published:** 2015-01-21

**Authors:** Masayuki Hamakawa, Takayuki Uozumi, Naoko Ueda, Yuichi Iino, Takaaki Hirotsu

**Affiliations:** Graduate School of Systems Life Sciences, Kyushu University, Fukuoka, 812-8581 Japan; Department of Biology, Faculty of Sciences, Kyushu University, Fukuoka, 812-8581 Japan; Department of Biophysics and Biochemistry, Graduate School of Science, The University of Tokyo, Tokyo, 113-0033 Japan; Department of Biology, Graduate School of Sciences, Kyushu University, 6-10-1 Hakozaki, Higashi-ku, Fukuoka 812-8581 Japan; Division of Applied Medical Sensing, Research and Development Center for Taste and Odor Sensing, Kyushu University, Fukuoka, 819-0395 Japan

**Keywords:** *C. elegans*, Exploratory behavior, Glutamate receptor, RNAi method, The Ras-MAPK pathway

## Abstract

**Background:**

The nematode worm *Caenorhabditis elegans*, in which loss-of-function mutants and RNA interference (RNAi) models are available, is a model organism useful for analyzing effects of genes on various life phenomena, including behavior. In particular, RNAi is a powerful tool that enables time- or cell-specific knockdown via heat shock-inducible RNAi or cell-specific RNAi. However, conventional RNAi is insufficient for investigating pleiotropic genes with various sites of action and life stage-dependent functions.

**Results:**

Here, we investigated the Ras gene for its role in exploratory behavior in *C. elegans*. We found that, under poor environmental conditions, mutations in the Ras-MAPK signaling pathway lead to circular locomotion instead of normal exploratory foraging. Spontaneous foraging is regulated by a neural circuit composed of three classes of neurons: IL1, OLQ, and RMD, and we found that Ras functions in this neural circuit to modulate the direction of locomotion. We further observed that Ras plays an essential role in the regulation of GLR-1 glutamate receptor localization in RMD neurons. To investigate the temporal- and cell-specific profiles of the functions of Ras, we developed a new RNAi method that enables simultaneous time- and cell-specific knockdown. In this method, one RNA strand is expressed by a cell-specific promoter and the other by a heat shock promoter, resulting in only expression of double-stranded RNA in the target cell when heat shock is induced. This technique revealed that control of GLR-1 localization in RMD neurons requires Ras at the adult stage. Further, we demonstrated the application of this method to other genes.

**Conclusions:**

We have established a new RNAi method that performs simultaneous time- and cell-specific knockdown and have applied this to reveal temporal profiles of the Ras-MAPK pathway in the control of exploratory behavior under poor environmental conditions.

**Electronic supplementary material:**

The online version of this article (doi:10.1186/s12915-015-0114-8) contains supplementary material, which is available to authorized users.

## Background

The nematode *Caenorhabditis elegans* is useful for studying the effects of a gene on behavior through facilitated behavioral analyses and well-developed genetic analyses. In this organism, various cell-specific promoters can be utilized for cell-specific expression to determine the function of a gene in a specific cell. Recently, temporal control of cell-specific expression using heat shock factor-1 (*hsf-1*) mutants has been reported [1]. In addition, the effects of gene knockdown can be assessed using various loss-of-function mutants, and researchers recently developed a new method of generating loss-of-function mutations in targeted genes in *C. elegans* [2].

RNAi is one of the most powerful tools for gene knockdown. RNAi-mediated cell-specific knockdown in *C. elegans* is a currently available technique [3] based on driving the expression of double-stranded RNA in target cells via cell-specific promoters. This method identifies the cells in which the target gene functions. Moreover, gene functions can be analyzed by this system because of cell-specificity, even if mutants of the gene show fatal phenotypes. In contrast to cell-specific promoters, heat shock promoters drive the expression of genes at arbitrary timing via heat shock [4]. A previous report has shown that RNA hairpins, which are driven by the heat shock promoter, induce knockdown of the target gene [5], suggesting the possibility of time-specific knockdown. However, heat shock promoters drive global expression, meaning that the knockdown is performed in the majority of cells, thereby removing vital cellular functions and thus inducing lethality when essential genes are knocked down by this method. Therefore, for detailed analysis of multifunctional genes, such as the components of the Ras-MAPK pathway described below, simultaneous time-specific and cell-specific knockdown is necessary. To our knowledge, however, such a method has not yet been developed in *C. elegans*.

Intracellular signal transduction pathways play an essential role in regulating diverse cellular functions. Most of these pathways have widespread functions in various tissues and at different stages. For example, the Ras-MAPK signaling pathway controls cell proliferation and differentiation at the embryonic stage [6-8] and regulates synaptic formation required for neural plasticity at the adult stage [9]. To distinguish and clearly understand the multiple functions of these signal transduction pathways, conditional knockdown systems, such as time- and cell-specific knockdown, are required.

The Ras-MAPK pathway in *C. elegans* plays a role in various processes, such as vulval induction, olfaction and germ-line apoptosis [10-12]. However, whether or not the signaling pathway is involved in the control of locomotion behavior is unknown. Animals use various strategies according to the circumstance in which they are placed to gain food efficiently. Under enriched environmental conditions in which animals are near food resources, efficient exploration is achieved by their use of perception, which incorporates olfaction, vision and touch. However, animals roam randomly to gain information when food resources are far from them. *C. elegans* has a simple nervous system. This worm is composed of only 302 neurons [13] and uses diverse strategies to obtain food. A recent study has shown that switching between dwelling and roaming activities according to the surroundings is controlled by a neural circuit and two neuromodulators, serotonin and neuropeptide pigment dispersing factor (PDF), which exhibit opposing effects [14]. In the neural circuit, serotonergic signaling initiates and extends the dwelling states, while PDF signaling affects roaming states by acting on each target neuron. While roaming, *C. elegans* uses two strategies to approach food resources efficiently in enriched environmental conditions. The first approach is klinotaxis (the weathervane strategy), in which the animals gradually curve according to the gradient of chemoattractants [15], and the second is klinokinesis (the pirouette strategy), in which they turn and change the direction of locomotion when a decrease in the concentration of chemoattractants is sensed [16].

The strategies for switching between dwelling and roaming activities, and those of approaching food under enriched environmental conditions, are well studied and understood. However, there is a scarcity of information regarding the strategy used by *C. elegans* when roaming under poor environmental conditions. Constitutive and exploratory head movements (termed foraging behavior) might be one possible strategy. IL1 and OLQ mechanosensory neurons are known to be involved in transducing signals during both aversive head-withdrawal and the control of foraging [17,18]. Further, of the motor neurons connecting to IL1 and OLQ neurons, RMD motor neurons possess the main synaptic projection [13], and thus play important roles in regulating head movements [17]. The neural circuit composed of IL1, OLQ and RMD neurons is essential for the regulation of spontaneous foraging and head-withdrawal movements. However, the role of foraging behavior on roaming and locomotion under poor environmental conditions, how head movements are regulated in foraging, and which signal transduction pathways are involved in the regulation of foraging are all unknown.

In this study, we analyzed the effect of the Ras-MAPK signaling pathway on the regulation of foraging behavior via an RNAi method with simultaneous time-specific and cell-specific knockdown of Ras. We found that mutants of the Ras-MAPK signaling pathway exhibit abnormal locomotion behavior (that is, a loopy pattern) under poor environmental conditions in which they are far from food resources. Cell-specific knockdown of the *ras* gene revealed that the Ras protein was functional in IL1, OLQ and RMD neurons, and thereby controlled foraging behavior. Further, results indicate that Ras regulated the localization of GLR-1 glutamate receptors in RMD neurons. Moreover, we demonstrated that the function of Ras at the adult stage was important for the regulation of GLR-1 localization in RMD neurons by using time- and cell-specific RNAi (T.C.RNAi). This method enabled cell-specific knockdown of gene functions at arbitrary timing.

## Results

### Mutations in the Ras-MAPK pathway lead to circular locomotion instead of normal exploratory foraging

**Figure 1:**
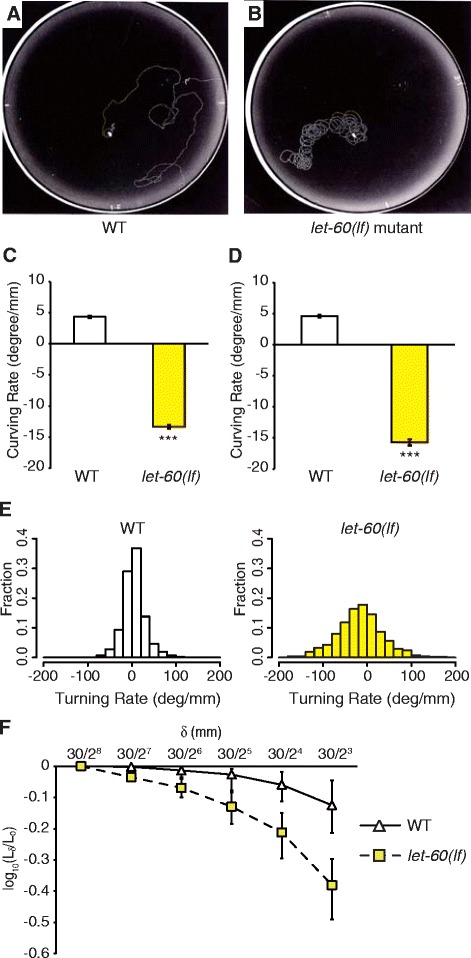
**Ras mutants exhibit abnormal locomotion behavior under poor circumstances. (A, B)** Tracks representative of wild type and the *let-60(n2021lf)* mutant in blank plates. The wild-type crawls around the field randomly, whereas the *let-60(lf)* mutant continues to move in a loopy pattern, termed circular locomotion (CL). **(C, D)** Average curving rate of wild type and *let-60(lf)* mutants for a fixed time (20 minutes) **(C)** or a fixed distance (30 mm) **(D)** of exploratory behavior (n ≥12,000 data points). Positive or negative values indicate curves toward ventral or dorsal sides, respectively. Asterisks indicate significant differences compared with wild-type animals (***P <10^−10^, Student’s *t*-test). **(E)** Histograms of curving rate in wild type and *let-60(lf)* mutants in the fixed distance (30 mm). **(F)** Quantitative analysis of directional locomotion. The median of log_10_(L_δ_/L_0_) at the distance of size δ in wild-type and *let-60(lf)* mutants is shown in log-log scale (n ≥30 animals) when analyzed in the fixed distance (30 mm). Error bars represent the first and the third quartile.

Under poor environmental conditions in which *C. elegans* is far from food resources, it crawled around the field to search for food resources. A previous study quantitatively characterized this behavior and reported that a locomotory behavior under these conditions has a long-range directionality and cannot be explained by a simple random isotropic model of locomotion [19]. Wild-type animals moved in an apparently unbiased manner when placed on a plate without any chemicals or food (Figure 1A). However, Ras mutants showed abnormal locomotion behavior on the blank plates. As the loss-of-function (lf) mutants of Ras, *let-60(lf)* [20,21] continued to move in a loopy pattern (Figure 1B), we named this phenotype ;circular locomotion’ (CL). We defined an animal that exhibited CL as one showing loopy tracks in more than 30% of the whole track left during the one hour test period (Table 1). A large proportion of *let-60(lf)* mutants were categorized with CL but exhibited milder phenotypes (see Additional file 1: Figure S1) compared to neuron-specific *let-60* RNAi strains described below, probably due to *n2021* being a weak allele. Ras and the downstream MAPK pathway are essential for numerous physiological cellular functions, and are highly conserved in diverse organisms [6-8]. Although the Ras-MAPK pathway in *C. elegans* regulates a number of processes [10-12], its involvement of the pathway controlling locomotion behavior is unknown.

**Table 1:** **Proportion of animals exhibiting circular locomotion (CL) in mutants of the Ras-MAPK pathway**

**Genotype**	**CL**	**Number**
Wild type	5.3%	64
*let-23(n1045)*	20.9%	42
*sem-5(n2019)*	22.9%	23
*let-60(n1046gf)*	8.5%	45
*let-60(n2021lf)*	58.4%	54
*lin-45(sy96)*	55.1%	41
*mek-2(n2678)*	49.6%	41
*mpk-1(ga117)*	49.7%	32

To analyze CL quantitatively, we measured the curving rate, which is defined as the change in direction of locomotion per unit length of an animal’s advancement [15], of the locomotion under poor conditions. Wild-type animals exhibited mostly random but slightly biased curves toward the ventral side (weakly positive value in Figure 1C). In contrast, *let-60(lf)* mutants exhibited significantly larger and directional curves toward the dorsal side (*P* <10^−10^) (Figure 1C). Given that each animal moved at a different velocity (thus moved for a different total distance), we calculated the average curving rate in the first 30 mm of each track. In this analysis, *let-60(lf)* mutants also exhibited significantly larger curves (*P* <10^−10^) than wild type (Figure 1D, E). These results suggest that CL may reflect large, directional curves.

In addition, to quantitatively measure the extent to which animals made directional tracks, we performed the analysis based on fractal analysis as described previously [19]. Briefly, the trajectory of each animal was divided into segments with length of size δ (mm), and the sum of linear distances of adjacent dividing points was computed as L_δ_ (mm). We then calculated L_δ_/L_0_ (A.U.), in which L_0_ (mm) indicates the length of the track. Again, the analysis was performed on the first 30 mm of each track, thus L_0_ = 30 mm. When animals tend to move in narrower areas, such as with CL, L_δ_/L_0_ is expected to decrease. L_δ_/L_0_ values for animals with *let-60(lf)* mutations tended to be lower than those of wild type animals (Figure 1F), indicating that the former tended to move in narrower areas than the latter. Taken together, CL is characterized as large, directional curves and in migration relatively narrow areas.

In *C. elegans*, components of the Ras-MAPK pathway: Ras, MAPKKK (Raf), MAPKK (MEK), and MAPK (ERK) are encoded by the *let-60*, *lin-45*, *mek-2* and *mpk-1* genes, respectively [20-27]. Each lf or null (0) mutant of the pathway, *let-60(lf)*, *lin-45(lf)*, *mek-2(0)* and *mpk-1(0)*, frequently exhibited CL (Table 1). Further, mutants of the upstream components of the signaling pathway, *let-23(lf)* and *sem-5(lf)* also exhibit CL (Table 1). The genes *let-23* and *sem-5* encode an EGF-receptor-family transmembrane tyrosine kinase and an adaptor protein orthologous to human GRB2, respectively [28,29]. However, *let-60(gf)* mutants (which carry the constitutive active form of LET-60Ras [30]) did not exhibit CL, suggesting that inactivation, and not hyperactivation, of the pathway caused CL. These results indicate that the Ras-MAPK pathway modulated locomotion behavior under poor environmental conditions in *C. elegans.*

We previously reported that the Ras-MAPK pathway controls both klinotaxis, a migratory behavior towards odorants, and olfactory plasticity [12,31,32]. AWC olfactory neuron- and AIY interneuron-specific expression of the *let-60* gene rescues the defects of klinotaxis and plasticity, respectively, of *let-60(lf)* mutants [31,32]. However, our results revealed that the expression of *let-60* in AWC and AIY did not reduce the number of the *let-60(lf)* mutants exhibiting CL (see Additional file 2: Table S1). *let-60(lf)* mutants have no abnormality in chemotaxis for sodium chloride [12]. We observed that mutants rarely exhibited CL when they moved towards the chemoattractants. A previous study reported that mutants exhibited moderate to severe defects in chemotaxis in response to 1 μl of 10^−2^, 10^−3^ and 10^−4^ dilutions of isoamyl alcohol on 9 cm plates (the start point of animals is 3 cm from the odorant) [31]. When mutants were placed 3 cm from 1 μl of a 10^−4^ or 10^−6^ dilution of isoamyl alcohol, the mutants exhibited CL frequently, as they were on plates without stimuli. In contrast, the proportion of those exhibiting CL significantly decreased (*P* <0.001) when mutants were exposed to 10^−2^ isoamyl alcohol (see Additional file 3: Figure S2). These results suggest that CL occurs independently of the defects of klinotaxis and olfactory plasticity.

### The Ras-MAPK pathway functions in neural circuits underlying foraging behavior to control direction of locomotion

**Figure 2:**
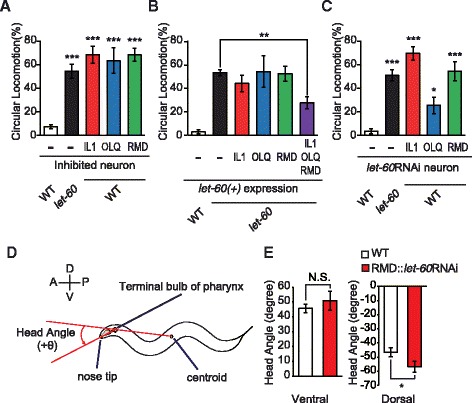
**LET-60 Ras in IL1, OLQ and RMD neurons controls the direction of locomotion. (A)** The proportion of animals exhibiting CL in wild type, *let-60(n2021lf)* mutants and transgenic animals in which IL1, OLQ or RMD neurons were inactivated by UNC-103(gf) (n ≥3 assays). **(B)** Rescue experiments in which the wild-type *let-60* gene was expressed in each or all the neurons regulating foraging in *let-60(lf)* mutants (n ≥3 assays). **(C)** IL1-, OLQ- or RMD-specific RNAi of *let-60* causes frequent CL (n ≥3 assays). **(D)** Definition of ‘head angle’ as the angle between two lines where one passes the worm centroid and the terminal bulb of pharynx and the other the terminal bulb of pharynx and nose tip. The ventral or the dorsal bends of the head are defined as positive or negative values, respectively. **(E)** Head angles of ventral and dorsal bends in WT and RMD::*let-60*RNAi animals. n ≥69 bends. Error bars represent SEM. Asterisks indicate significant differences compared with wild-type animals **(A, C, and E)** or *let-60(lf)* mutants **(B)** (*P <0.05, **P <0.01, ***P <0.001, Dunnett’s test or Student’s *t*-test). N.S., not significant. CL, circular locomotion; SEM, standard error of the mean; WT, wild type.

To elucidate the mechanism underlying impairment of the Ras-MAPK pathway-mediated CL, we analyzed the locomotion behavior of *let-60(lf)* mutants in more detail. *let-60(lf)* mutants exhibited abnormal foraging behavior in which their head movements were slightly asymmetrical and over-bent. This observation led us to assess whether abnormal foraging behavior induced CL. Previous reports have shown that the rate of spontaneous foraging is regulated by a neural circuit composed of three classes of neurons: IL1, OLQ, and RMD [17,18]. IL1 and OLQ are mechanosensory neurons, and RMD are motor neurons that receive main connections from IL1 and OLQ neurons, and innervate the head muscles [13]. Therefore, we examined whether blocking the activity of these neurons causes CL. *unc-103(gf)* was used to inhibit the activity of these neurons. This gene encodes a constitutively active form of the potassium channel UNC-103 and can reduce the neuronal activity by hyperpolarization [33,34]. We found that IL1, OLQ or RMD neuron-specific expression of *unc-103(gf)* in wild-type animals significantly (*P* <0.00001) caused CL (Figure 2A), indicating that inhibition of these neurons induced locomotion behavior in a loopy pattern in *C. elegans*, even if one class of neuron was impaired. A previous report indicated that the *osm-9* promoter we utilized for the expression in OLQ also drives the expression in AWA and ADL sensory neurons [35]. However, AWA- or ADL-ablated animals did not exhibit CL (see Additional file 4: Figure S3), indicating the importance of OLQ in the regulation of locomotion. These results suggest that the neural circuit (that is, IL1, OLQ and RMD neurons) involved in foraging behavior played an essential role in determining the direction of locomotion.

These results led us to investigate whether the Ras-MAPK pathway plays a role in the neural circuit involved in foraging behavior to control locomotion. We, therefore, conducted rescue experiments where the wild-type *let-60* gene (*let-60*(+)) was expressed in neurons regulating foraging behavior in *let-60(lf)* mutants. We found that expression of *let-60*(+) in all three neuronal classes rescued the CL phenotype in *let-60(lf)* mutants, which was not found when the expression of *let-60(+)* occurred in a single class of neurons (Figure 2B). These results suggested that LET-60 Ras functions in all classes of neurons, which regulate foraging behavior. Further, this suggestion was consistent with the finding that impairment of *let-60* in a single class of neurons caused CL (Figure 2C), as described below.

We next performed cell-specific knockdown experiments using RNAi [3]. Cell-specific knockdown of the *let-60* gene in each class of neurons (IL1, OLQ, or RMD) caused CL (Figure 2C). In particular, knockdown of *let-60* in IL1 or RMD neurons strongly (*P* <0.00001) induced CL, suggesting that LET-60 Ras largely contributed to the function of IL1 and RMD neurons. Taken together, these results indicate that functions of LET-60 Ras in all IL1, OLQ and RMD neurons were required for foraging behavior to control the direction of locomotion. Compared to animals with inhibition of neurons, neuron-specific *let-60* RNAi strains exhibited milder phenotypes (see Additional file 1: Figure S1), suggesting that pathways other than Ras signaling might also be involved in the regulation of locomotion, although the possibility that RNAi is at least somewhat effective cannot be excluded.

We directly observed and quantified foraging behavior in wild type and animals with RMD-specific RNAi knockdown of *let-60*. We monitored the head angle of worms during locomotion on plates without any chemicals or food. This angle was defined as that between two lines in which one passes the worm centroid and the terminal bulb of pharynx and the other the terminal bulb of pharynx and nose tip (Figure 2D). Ventral bends were counted as positive and dorsal bends as negative values, respectively (Figure 2E). The mean head angle of dorsal bends in animals with RMD-specific *let-60* RNAi was significantly (*P* <0.05) larger than in wild type animals, while that of ventral bends was not (Figure 2E). This result seems to be consistent with the finding that *let-60(lf)* mutants tended to exhibit dorsal-biased large turns in exploratory behavior (Figure 1C, D) and indicates that Ras in RMD neurons is important for foraging behavior, suggesting a correlation between foraging and exploratory behavior.

### The Ras-MAPK pathway regulates the localization of glutamate receptors in RMD neurons

We next explored the role of the Ras-MAPK pathway in IL1, OLQ and RMD neurons. The Ras-MAPK pathway is essential for neurogenesis in fruit flies and mice [36,37]. To clarify if this pathway was required for neurogenesis of these neurons, we observed the morphology of the neurons in *let-60(lf)* mutants. These mutants did not exhibit any apparent morphological defects in IL1, OLQ or RMD neurons (see Additional file 5: Figure S4), suggesting that the Ras-MAPK pathway was not involved in the morphogenesis of these neurons.

The Ras-MAPK pathway also has important roles in synapse formation and the control of synaptic protein localization [38-42]. To examine whether this pathway regulates formation of pre-synapses in IL1 and OLQ neurons, we observed the localization of synaptobrevin, SNB-1 [43], in the axons of these neurons. SNB-1 is a synaptic vesicle protein essential for vesicle docking or fusion, which is required for synaptic transmission [44,45]. We observed that SNB-1 localization was not affected by IL1- or OLQ-specific knockdown of *let-60* (see Additional file 6: Figure S5). Therefore, the Ras-MAPK pathway might not be involved in presynaptic formation of these neurons.

**Figure 3:**
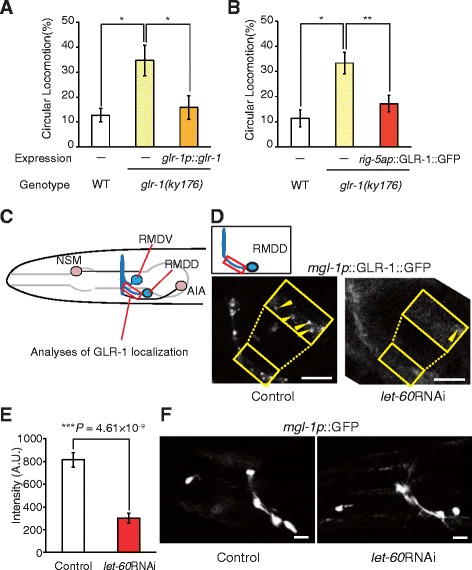
**LET-60 Ras mediates the localization of GLR-1 glutamate receptors in RMD neurons. (A)** The proportion of wild type, *glr-1(ky176)* mutants and *glr-1* mutants with expression of *glr-1* gene by its own promoter exhibiting CL (n ≥5 assays). **(B)** The proportion of wild type, *glr-1(ky176)* mutants and *glr-1* mutants with expression of GLR-1::GFP by *rig-5a* promoter exhibiting CL (n ≥4 assays). Error bars represent SEM. Asterisks indicate significant differences (*P <0.05, **P <0.01, Student’s *t*-test). **(C)** *mgl-1* promoter drives expression in NSM, RMDV, RMDD and AIA neurons. Neurites of RMDD neurons (enclosed by a red rectangle) were investigated by analyzing the clusters of GLR-1::GFP in RMD neurons specifically. **(D)** GLR-1::GFP clusters expressed by the *mgl-1* promoter in wild type (left panel) and wild type with RMD-specific RNAi of *let-60* (right panel). Enlarged images show neurites of RMDD neurons (yellow arrowheads = GLR-1::GFP clusters). **(E)** Average intensity of GLR-1::GFP clusters in neurites of RMDD neurons is significantly reduced by RMD-specific knockdown of *let-60* (n ≥10 animals). Error bars represent SEM. An asterisk indicates significant differences (Student’s *t*-test). **(F)** Representative images of *mgl-1p*::GFP in wild type (left panel) and wild type with RMD-specific RNAi of *let-60* (right panel). Scale bars = 10 μm. CL, circular locomotion; SEM, standard error of the mean.

Previous studies have shown that Ras signaling drives the synaptic delivery of α-amino-3-hydroxy-5-methyl-4-isoxazolepropionic acid (AMPA) receptors in post-synapses [40,42]. In *C. elegans,* the *glr-1* gene (which encodes an AMPA-type ionotropic glutamate receptor) is expressed in neurons, including RMD [17,46]. Our results revealed that *glr-1* mutants also displayed CL (Figure 3A). The expression of *glr-1* by its own promoter restored the defect in locomotion behavior of *glr-1* mutants (Figure 3A). Moreover, RMD-specific expression of GLR-1::GFP significantly (*P* <0.01) rescued the CL phenotype of *glr-1* mutants (Figure 3B). These results suggest that GLR-1 in RMD neurons played an important role in controlling the direction of locomotion and also indicated that GLR-1::GFP was functional. We next investigated whether the Ras-MAPK pathway was associated with GLR-1 for the regulation of locomotion behavior. To assess the effect of this pathway on GLR-1, we investigated the localization of GLR-1::GFP in neurites of RMD neurons in wild-type and animals with RMD-specific knockdown of *let-60* via fluorescence. GLR-1::GFP was expressed by *mgl-1* promoter, which drives the expression in NSM, AIA and RMD neurons [47]. Distinguishing the neurites of RMD and AIA neurons in the nerve ring is difficult. Thus, we targeted the neurites of RMDD neurons because these neurons are distinct from those of AIA (Figure 3C). In wild-type animals, punctate localization of clustered GLR-1 was observed in neurites of RMD neurons (Figure 3D). However, in RMD neurons of RMD-specific *let-60* knockdown animals, GLR-1::GFP clusters were very faint (43.8%) or not detected (56.2%) (Figure 3D, E). We confirmed that the expression of GFP by the *mgl-1* promoter was not affected by the knockdown of the *let-60* gene (Figure 3F), suggesting that LET-60 Ras did not act on the *mgl-1* promoter. These results indicated that Ras was required for normal localization of GLR-1 in post-synapses of RMD neurons.

### Time- and cell-specific RNAi is useful for analyses of the temporal profile of gene functions in the specific cell

We then analyzed the manner in which the Ras-MAPK pathway modulated the localization of GLR-1. The Ras-MAPK pathway is involved in morphogenesis, synaptic formation, and neural plasticity [36-42]. Therefore, our results led us to test two hypotheses: (1) the Ras-MAPK pathway plays a role in the development of synapses or synaptic connections of RMD neurons at the embryonic stage, and (2) the signaling pathway is involved in GLR-1 localization machinery at the adult stage. To examine these hypotheses, we analyzed the stage at which LET-60Ras mediated the control of GLR-1 localization in RMD neurons.

To dissect the function of a gene which has different roles according to the stages, such as Ras, a method to regulate the function of the gene spatially and temporally is needed. RNAi is one of the powerful tools for gene knockdown, and a recent study has introduced the cell-specific knockdown technique using RNAi [3]. The method of cell-specific knockdown is based on the expression of double-stranded RNA by the cell-specific promoter (see Additional file 7: Figure S6A) [3]. However, this method is incapable of performing time-specific knockdown and dissecting the stage at which the target protein functions in a specific cell. To perform simultaneous time-specific and cell-specific knockdown, we modified the previous method [3]. The heat shock promoter *hsp16-2* [4], which drives the expression in almost all tissues, is able to activate gene expression at arbitrary timing by heat shock. By introducing the heat shock promoter to the cell-specific RNAi technique, we established a new method for time-specific and cell-specific knockdown of genes, in which expression of one RNA strand (sense or anti-sense) was driven by a cell-specific promoter, and the expression of the other RNA strand was induced by a heat shock promoter (see Additional file 7: Figure S6B). Under normal conditions (20°C to 24°C), single stranded RNA was expressed in the target cells by the cell-specific promoter, whereas under the heat shock-inducing condition (30°C to 33°C), double-stranded RNA was expressed in only target cells by the heat shock promoter and the cell-specific promoter. Overall, this new technique was expected to elicit simultaneous time-specific and cell-specific knockdown by heat shock. Therefore, this method was termed time-specific and cell-specific RNAi (T.C.RNAi).

To confirm the effect of the T.C.RNAi method, we examined whether expression of GFP can be decreased in the time and cell-specific manner by GFP T.C.RNAi. To clearly observe the effect of RNAi, we photobleached pre-existing GFP, which otherwise has a relatively long half life [48], before T.C.RNAi and observed the recovery of GFP fluorescence after T.C.RNAi. This way we can assess the decrease of mRNA abundance caused by RNAi. To quantitatively compare the GFP intensity before and after T.C.RNAi, we measured the GFP intensity in the same individuals before photobleaching (intact), just after photobleaching and after a heat shock for 30 minutes at 33°C and a recovery for one hour at 20°C. Then we calculated the rate of change in the fluorescence intensity of each animal. Based on this rate, the recovery ratio of GFP intensity compared to controls (without heat shock) was measured (see Methods).

**Figure 4:**
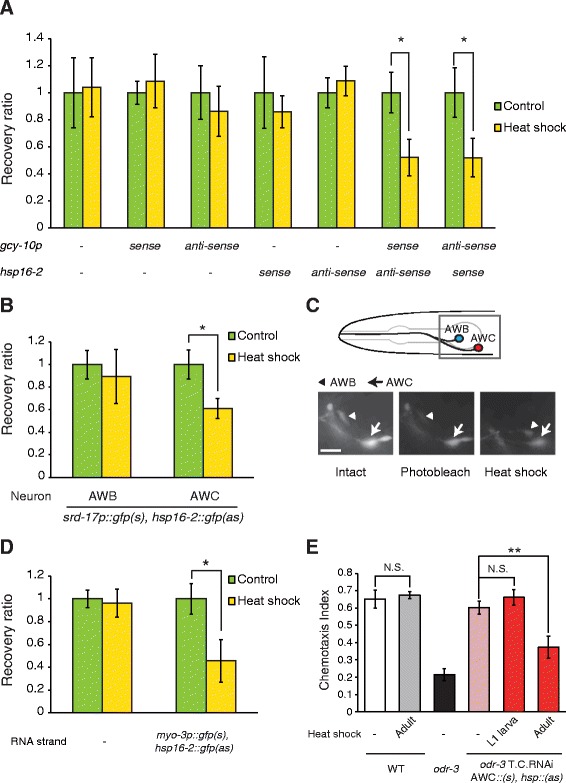
**Confirmation of the effect of the novel method, time- and cell-specific RNAi (T.C.RNAi). (A)** The average recovery ratio of GFP intensity of AWC neurons in animals without RNAi constructs, animals with only *gcy-10p*::*gfp(s), gcy-10p*::*gfp(as), hsp16-2*::*gfp(s)*, *hsp16-2*::*gfp(as)*, animals with both *gcy-10p*::*gfp(s)* and *hsp16-2*::*gfp(as)* or both *hsp16-2*::*gfp(s)* and *gcy-10p*::*gfp(as)* after heat-shocked or control condition. (n ≥9 animals). **(B)** The average recovery ratio of GFP intensity of AWB and AWC neurons in animals with *srd-17p*::*gfp(s)* and *hsp16-2*::*gfp(as)* after heat-shocked or control condition (n ≥10 animals). **(C)** Representative images of *gcy-10p*::GFP in animals with *srd-17p*::*gfp(s)* and *hsp16-2*::*gfp(as)* before (left panel, intact) and after (center panel, photobleach) photobleaching and after heat-shocked condition (right panel, heat shock). Arrow heads indicate AWB neurons, and arrows indicate AWC neurons. Scale bars = 10 μm. **(D)** The average recovery ratio of GFP intensity of anterior or lateral protrusions of vulva in animals without RNAi constructs or animals with *myo-3p*::*gfp(s)* and *hsp16-2*::*gfp(as)* after heat-shocked or control condition (n ≥14 protrusions of vulva). **(E)** Chemotaxis to isoamyl alcohol (1:1000) in wild-type animals, *odr-3* mutants and wild-type animals with *odr-3* T.C.RNAi (n ≥4 assays). AWC::(*s*) and *hsp*::(*as*) mean *gcy-10p*::*odr-3(s)* and *hsp16-2*::*odr-3(as)*. Error bars represent SEM and asterisks indicate significant differences (*P <0.05, **P <0.01, Dunnett’s test or Student’s *t*-test). SEM, standard error of the mean.

We first analyzed the effect of GFP T.C.RNAi in AWC sensory neurons. GFP was expressed by the *gcy-10* promoter which drives the expression in AWC, AWB and I1 [49] and monitored the GFP intensity in cell bodies of AWC. In adult animals expressing both *gcy-10::gfp(s)* and *hsp::gfp(as)*, the recovery ratio of GFP intensity in AWC significantly decreased after the heat shock compared to that after the mock treatment (Figure 4A). The expression of double-stranded RNA by the reciprocally exchanged promoter also induced GFP knockdown after the heat shock (Figure 4A). The GFP intensity in AWC was normally recovered after the heat shock in animals without expression of the RNAi constructs, suggesting the decrease of the recovery ratio was not due to an influence of heat shock on the *gcy-10* promoter itself (Figure 4A). We also confirmed that expression of only a single RNA strand driven by a heat shock promoter or a cell-specific promoter could not decrease the recovery ratio (Figure 4A).

Moreover, to verify the cell-specificity of the gene knockdown by T.C.RNAi, we monitored GFP intensity in both AWC and AWB neurons at the same time. We used *gcy-10* promoter for the expression of GFP in both AWC and AWB neurons, and *srd-17* promoter for the expression of a single-stranded RNA specifically in AWC neurons. In animals that expressed *srd-17p*::*gfp(s)*and *hsp*::*gfp(as)*, recovery of GFP intensity in AWC was reduced but not in AWB after the heat shock (Figure 4B, C), indicating the cell-specific effect of T.C.RNAi. Taken together, T.C.RNAi could induce time- and cell-specific knockdown of GFP in AWC neurons effectively.

Next, to investigate whether T.C.RNAi could cause gene knockdown in tissues other than neurons, we performed GFP knockdown in vulval muscles. We found a decrease of the recovery ratio of GFP intensity in vulval muscles after heat shock treatment, but not after control treatment in animals with *myo-3*::GFP, *myo-3*::*gfp(s)* and *hsp*::*gfp(as)* (Figure 4D). We confirmed that GFP intensity in animals without expression of RNAi constructs was recovered normally after the heat shock (Figure 4D). These results suggested that T.C.RNAi could cause gene knockdown in muscles as well as neurons in a cell-specific manner.

In addition, to assess whether T.C.RNAi can be applied to endogenous genes, we performed knockdown of *odr-3* by T.C.RNAi. The *odr-3* gene encodes a G protein α which mainly functions in AWC chemosensory neurons and is essential for olfactory responses to odorants, including isoamyl alcohol, which is sensed by AWC [50]. We found that animals in which *odr-3* was knocked down in AWC neurons by T.C.RNAi at the adult stage showed a defect in the response to isoamyl alcohol (Figure 4E). However, the adult transgenic animals that had undergone heat shock treatment at the L1 larval stage exhibited a normal response (Figure 4E). These results indicated that ODR-3 worked in AWC neurons at the adult stage, which is consistent with the well-known function of ODR-3 in the olfactory signaling pathway. These results suggested that T.C.RNAi could induce time- and cell-specific knockdown of endogenous genes as well as transgenes, and the possibility that our new method could be applied widely.

### T.C.RNAi revealed the stage at which Ras functions in RMD neurons to control locomotion behavior

**Figure 5:**
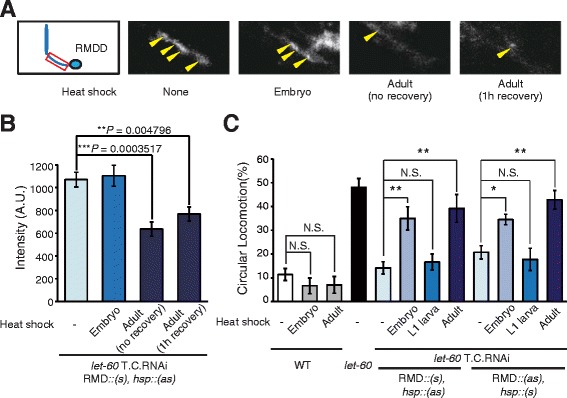
**T.C.RNAi provides detailed profiles of LET-60Ras in RMD neurons. (A)** Representative images of GLR-1::GFP in neurites of RMDD of transgenic animals with *let-60* time- and RMD-specific RNAi (T.C.RNAi) (rectangle = the neurite of RMDD; yellow arrowheads = GLR-1::GFP clusters). **(B)** Average intensity of GLR-1::GFP fluorescence in RMDD neurons of transgenic animals with *let-60* T.C.RNAi after heat shock at the embryonic or adult stage (n ≥10 animals). **(C)** The proportion of wild type, *let-60(lf)* mutants, and animals with *let-60* T.C.RNAi after heat shock treatment exhibiting CL (n ≥3 assays). In **(B)** and **(C)**, RMD::(*s*) and *hsp*::(*as*) mean *rig-5ap*::*let-60(s)* and *hsp16-2*::*let-60(as)*, respectively. Error bars represent SEM and asterisks indicate significant differences (*P <0.05, **P <0.01, ***P <0.001, Dunnett’s test or Student’s *t*-test). SEM, standard error of the mean.

Using our new method, T.C.RNAi, we investigated the effects of *let-60* knockdown on the localization of GLR-1 in RMD neurons at the embryonic and adult stage. Our results indicated that GLR-1 localization was not affected by *let-60* knockdown at the embryonic stage (Figure 5A, B). However, knockdown of *let-60* at the adult stage caused significant abnormality in the localization of GLR-1 (Figure 5A, B), which was the same phenotype induced by cell-specific RNAi (Figure 3D, E). We confirmed that the *rig-5a* cell-specific promoter also drove RMD-specific expression at the embryonic stage (see Additional file 8: Figure S7). These results indicated that in RMD neurons at the adult stage, the Ras-MAPK pathway controlled GLR-1 localization.

We next analyzed the stage at which the Ras-MAPK pathway in RMD neurons regulated locomotion behavior. The knockdown of *let-60* in RMD neurons at the L1 larva stage resulted in no defects in locomotion behavior, whereas the expression of *let-60* double-stranded RNA at the adult stage significantly (*P* <0.01) increased the rate of CL (Figure 5C), indicating that Ras regulated locomotion at the adult stage in RMD. The same result was obtained even when the sense and the anti-sense strand were expressed by the reciprocally changed promoter (Figure 5C). These results were consistent with the earlier finding that Ras in RMD neurons at the adult stage was necessary for the normal localization of GLR-1 (Figure 5A, B). However, knockdown of *let-60* at the embryonic stage also significantly induced CL (Figure 5C), suggesting a role for Ras other than the regulation of GLR-1 localization at the embryonic stage to control locomotion behavior. Neither wild-type animals exposed to heat shock nor transgenic animals without heat shock showed CL (Figure 5C). Further, we confirmed that expression of only a single RNA strand by a heat shock promoter or a cell-specific promoter cannot cause knockdown of the target gene (see Additional file 9: Figure S8).

## Discussion

In the present study, we developed a novel method, T.C.RNAi that achieves simultaneous time- and cell-specific knockdown. We confirmed that T.C.RNAi causes time- and cell-specific knockdown of GFP. We further show that T.C.RNAi caused abnormal locomotion behavior and mislocalization of GLR-1 by adult stage- and RMD neurons-specific knockdown of *let-60Ras*, and aberrant responses to an odorant by adult stage- and AWC neurons-specific knockdown of *odr-3* via heat shock. Temporal and spatial restriction of the effects of knockdown is necessary for the analysis of various and crucial genes, such as Ras. LET-60Ras determines the fate of the excretory duct cell, which is required for osmoregulation [11]. Moreover, null mutants are lethal in the L1 larval stage [11]. Nevertheless, in the present study, the effects of *let-60* knockdown at both the embryonic and L1 larval stage was observed by T.C.RNAi without lethality. Overall, T.C.RNAi might provide a basis for understanding temporal and special profiles of essential genes. This approach was used in the present study for Ras, which is involved in various physiological activities, such as development, signal transduction and neural plasticity.

We analyzed the exploratory behavior of *C. elegans*. Under enriched conditions, *C. elegans* exhibited klinokinesis and klinotaxis (a change in the direction of locomotion to approach food by turning and curving, respectively, according to the gradient of chemoattractants). Our results indicated that in contrast to these responses, foraging behavior was used by *C. elegans* to search for food resources over wide-ranging areas and determined the direction of locomotion under poor conditions. We found that CL occurred in *let-60(lf)* mutants under poor conditions, whereas the approach to sodium chloride of the mutants was normal [12]. Therefore, *C. elegans* may exhibit foraging behavior to search for food cues specifically under poor environmental conditions. Moreover, once animals detect food stimuli, the possible contribution of klinokinesis and klinotaxis may play a greater role than foraging to determine both locomotion direction and the movement towards food resources.

Our results showed that the Ras-MAPK pathway was functional in IL1, OLQ and RMD neurons. In IL1 and OLQ neurons, *let-60* knockdown did not cause abnormal localization of SNB-1, suggesting roles other than the regulation of synaptic vesicle localization for the Ras-MAPK pathway. In pre-synapses, this pathway controls the localization of transporter vesicles, in addition to other roles [38]. A previous study showed that MAPK modulates the release probability at glutamatergic synapses in rats [51]. In *C. elegans*, *eat-4* encodes the ortholog of BNPI vesicular glutamate transporter that appears to have a role in the synthesis of glutamate or concentrate glutamate into synaptic vesicles, and *eat-4* is expressed in IL1 and OLQ neurons [51-53]. We previously reported that PKC-1 which controls synaptic transmission functions downstream of Ras signaling in AWC neurons in which glutamate is released as a neural transmitter and it is mediated by EAT-4 [31]. In the present study, we found that CL occurred in mutants of *eat-4* (see Additional file 10: Table S2). Therefore, the Ras-MAPK pathway may be associated with the regulation of EAT-4 directly via phosphorylation, or indirectly via other molecules, including transcription factors in IL1 and OLQ neurons to control foraging behavior.

In RMD neurons, our findings show that LET-60Ras plays an essential role in the localization of GLR-1. Although *lin-1* or *lin-31* encodes a transcription factor that functions downstream of MAPK in vulval induction [11], the MAPK signaling pathway unlikely activates these transcription factors to control the expression of GLR-1, because our current findings from T.C.RNAi experiments indicated that GLR-1 mislocalization was induced within 30 minutes by heat shock at the adult stage. That MAPK might directly phosphorylate and regulate GLR-1 or the proteins required for its localization is possible. A recent report shows that auxiliary subunits are important for regulating AMPA receptor trafficking to the synaptic membrane [54]. Further, the cytoskeletal protein, α-adducin-1 (which co-localizes with GLR-1 in post-synapses to control GLR-1 dynamics), is expressed in RMD neurons [55]. Our present results from the T.C.RNAi analyses show that Ras in RMD neurons at the embryonic stage is required for the regulation of foraging, but not for control of GLR-1 localization. These findings show that LET-60Ras was neither involved in morphogenesis of RMD neurons nor formation of post-synapses, both of which are necessary for the normal localization of GLR-1 in RMD. Therefore, we speculate that LET-60 Ras might regulate the formation of, or play a role at, the neuromuscular junction in RMD motor neurons during the embryonic stage. A previous study has shown that the Ras-MAPK pathway is triggered by trans-synaptic signaling, and is responsible for the formation of the neuromuscular junction and its functions [56]. In *C. elegans*, the acetylcholine transporter and choline acetyltransferase play a role at the neuromuscular junction and are encoded by *unc-17* [57] and *cha-1* [58], respectively. Further, these two genes are localized in the synaptic region [59]. Therefore, exploring the functional role of UNC-17 and CHA-1 in RMD neurons may shed more light on the function of the Ras-MAPK pathway at the embryonic stage in RMD neurons.

## Conclusions

Our results demonstrate that the Ras-MAPK pathway functions in the neural circuit underlying the foraging behavior essential for the control of the direction of locomotion under poor environmental conditions in *C. elegans*. The pathway regulates localization of GLR-1 glutamate receptors in RMD neurons. We established a time- and cell-specific RNAi method which demonstrated the temporal profile of the signaling pathway in specific neurons. The control of GLR-1 localization in RMD requires Ras at the adult stage. This method can be applied to other genes.

## Methods

### Strains and culture

Strains were cultured on NGM plates (at 20°C, under standard conditions) with *Escherichia coli* NA22 as the food source [60]. The strains used in this study were: wild-type Bristol strain N2, *let-60(n2021lf)*, *let-60(n1046gf)*, *lin-45(sy96)*, *mek-2(n2678)*, *mpk-1(ga117)*, *let-23(n1045)*, *sem-5(n2019)*, *odr-3(n2150)*, *glr-1(ky176)* and *eat-4(ky5)*.

### Plasmid construction and germ-line transformation

To drive cell-specific expression, we used three types of promoters: (1) IL1-specific *aqp-6* promoter [61], (2) OLQ and two other sensory neuron-specific *osm-9* promoters [35], and (3) RMD-specific *rig-5a* promoter [62]. The *mgl-1* promoter [47], which drives the expression in NSM, RMDD, RMDV and AIA, was used for analyses of GLR-1 localization in RMD neurons. These promoters were amplified by PCR with two primers from genomic DNA:

- *aqp-6* promoter: forward primer (FP) = 5′-ctttattcattttgcaattga-3′, Reverse primer (RP) = 5′-tttcggaa caatatctgaact-3′
- *osm-9* promoter: FP = 5′-agcctaaaaaacagtgag-3′, RP = 5′-gtttggtttctgaaaaaa-3′
- *rig-5a* promoter: FP = 5′-attacttgtacatttcca-3′, RP = 5′-tgatggttgttgaattg-3′
- *mgl-1* promoter: FP = 5′-gattttgcagaacttgga-3′, RP = 5′-tatttcgcgatttttttc-3′.

GLR-1::GFP was constructed as previously reported [63]. GFP, Venus, SNB-1::RFP [64], GLR-1::GFP and *unc-103(gf)* [34] were inserted downstream of the above promoters. *glr-1* cDNA was amplified with the template, ORFeome clone AAA92006 (Open Biosystems, Lafayette, Colorado, USA), and connected to its own promoter [46]. AWC olfactory neuron-specific expression of *let-60* was driven by the *gcy-10* [49] and AIY interneuron-specific expression by the *ttx-3* promoter [65]. Transgenic lines were generated by microinjection as previously described [66]. *lin-44p*::GFP and *myo-3p*::GFP were used as transformation markers.

### Exploratory behavior

Exploratory behavior was observed using assay plates without stimuli, as shown in Figure 1. Plates were desiccated before the assays to visualize the tracks of animals. After worms were placed at the starting point and allowed to crawl for one hour, we counted the number of worms that moved continuously only in a loopy pattern (strong), exhibited both normal and loopy patterns (the latter covering ≥30% of the full tracks) (mild) and exhibited normal trajectories (<30% of loopy tracks) (normal). We defined animals exhibiting CL as those for which ≥30% of the full tracks were loopy (strong and mild). Loopy trajectories were defined as parts of tracks forming closed circles with a diameter of less than 1 cm. We then calculated the proportion of all worms exhibiting CL out of 10 counted per assay. The diameter of the assay plates was 9 cm. The composition of the medium was 20 g/l agar, 5 mM KPO_4_, 1 mM CaCl_2_ and 1 mM MgSO_4_.

### Quantitative analysis of circular locomotion

To quantitatively analyze exploratory behavior, we used the multi worm tracking system [67], which simultaneously tracks the behavior of four to five worms on assay plates for 20 minutes. The interval between image capture was 1,000 ms. We excluded data of animals that crossed each other. Curving rate was defined as the change in direction of locomotion per unit length of an animal’s advancement and was calculated as previously described [15].

To quantitatively measure the extent to which animals made directional paths, we performed the analyses based on fractal analysis as previously described [19]. We constructed a set of trajectories (termed ‘coarse-grained trajectories’ in the previous study) by dividing the trajectory into sub-trajectories with a length of size δ and making linear connections between end points of sub-trajectories. Given that each animal moved at a different velocity, we used trajectory lengths instead of time intervals. We calculated the lengths of coarse-grained trajectories as L_δ_ and calculated L_δ_/L_0_, in which L_0_ represents the total length of each original track.

### Analysis of foraging

To quantify the foraging behavior, we measured the head angle of animals mobile on plates without any chemicals or food. We recorded the exploratory behavior of each animal on camera and measured the coordinates of the worm centroid, the terminal bulb of pharynx and the nose tip. The head angle was defined as the angle between two lines, one connecting the worm centroid and the terminal bulb of pharynx, and the other the terminal bulb of pharynx and nose tip (Figure 2D). Ventral bends were counted as positive values and dorsal bends as negative values. The head angle was calculated every 500 ms.

### Circular locomotion phenotype with odorant signals

*let-60(lf)* mutant animals were placed 3 cm from 1 μl of 10^−2^, 10^−4^ or 10^−6^ dilutions of isoamyl alcohol. After 30 minutes, the number of worms exhibiting circular locomotion was counted.

### Chemotaxis assay

Chemotaxis assays were performed, as previously described [68]. The chemotaxis index was calculated as previously described [68]. A 10^−3^ dilution of isoamyl alcohol was used as an attractive odorant.

### Cell-specific knockdown of *let-60*

The cell-specific knockdown of *let-60* was performed, as previously described [3]. The target region of *let-60* was amplified with primers: Tf = 5′-aaatccttctccacttcgttttc-3′, Tr = 5′-aagaggatcgatcacaagtttca-3′. The *aqp-6*, *osm-9* or *rig-5a* promoter was used to drive the specific expression in IL1, OLQ or RMD, respectively.

### T.C.RNAi

The target region of *let-60* was amplified with primers described in the aforementioned section. Amplification of *odr-3* was performed with Tf = 5′-ctcatgccagagcaatgaaa-3′, Tr = 5′-atgcgtttgctctctcaggt-3′. The target region of GFP was amplified with the primers Tf = 5′-atgagtaaaggagaagaact-3′ and Tr = 5′-ctatttgtatagttcatcca-3′. Amplified target regions were inserted downstream of each cell-specific promoter or the heat shock promoter *hsp16-2*. Transgenic animals used in T.C.RNAi experiments have two DNA fragments, one of which drives the expression of a sense strand of the target region by the cell-specific promoter, and the other by the expression of an anti-sense strand by the heat shock promoter. For locomotion behavior analysis, the sense and the anti-sense strand were also expressed by the reciprocally changed promoter. To drive AWC-specific expression, we used the *gcy-10* promoter [49] and the *srd-17* promoter [69]. The *gcy-10* promoter was amplified with primers FP = 5′-tgggtacaacaatttctc-3′, RP = 5′-ataattggccttctgctcaaa-3′, and the *srd-17* promoter was amplified with primers FP = 5′- ccgtctaacttctttttg −3′, RP = 5′-tattgaattggcaaatgg-3′ by polymerase chain reaction for the analysis of chemotaxis behavior and the analysis of GFP knockdown in sensory neurons. The *myo-3* promoter, which was amplified with primers FP = 5′- ttgaataaaataattttccc −3′, RP = 5′-tggatctagtggtcgtgggt-3′ by polymerase chain reaction, was used for the analysis of GFP knockdown in vulva.

### GFP knockdown by T.C.RNAi

In the analysis of GFP knockdown, before heat shock treatment, GFP was photobleached by 10-fold stronger excitation light than that for observation for 25 seconds or 45 seconds for sensory neurons or vulval muscles, respectively. Such photobleaching decreased the intensity of GFP to 50% to 70% of the intact intensity. Then, the animals were transferred to an 0.2 ml tube with basal buffer (0.5 g/L gelatin, 5 mM KPO_4_, 1 mM CaCl_2_ and 1 mM MgSO_4_). This tube was incubated under heat-shocked conditions at 33°C for 30 minutes or under control conditions at 20°C for 30 minutes, and animals in the tube were placed on the NGM plate with food at 20°C for one hour for the recovery. We recorded GFP fluorescence three times, before and after photobleaching, and after the recovery from heat shock at the same intensity of the excitation light. We monitored GFP intensity in cell bodies of AWC and AWB neurons, and in anterior or lateral protrusions of vulva using the Leica digital microscope DMI3000B with a 40x objective lens. The intensity of GFP was measured based on the region of interest (ROI), the size of which was equivalent in all animals used for these analyses. The average intensity at three random background points was calculated as the background intensity and was subtracted from the intensity of GFP. We calculated the rate of change in the GFP intensity of each animal as ((GFP intensity after heat shock) – (GFP intensity after photobleaching))/((intact GFP intensity) – (GFP intensity after photobleaching)). To quantify and normalize the recovery of the intensity after heat shock treatment, the recovery ratio was calculated as ((the rate of change in the GFP intensity of each animal after the heat shock) – (the average intensity change of control treatment) + 1).

### Heat shock treatment

For the embryonic stage, adult cuticles were dissolved in lysis solution (NaOH: NaClO = 3: 4) to gain eggs. The eggs were incubated under heat shock at 30°C for eight hours and then shifted to the standard condition (20°C) and cultivated to adulthood. For the L1 larval stage, eggs were kept under standard conditions for 20 hours after lysis treatment. The eggs were then shifted to the heat shock at 30°C for 16 hours and switched to the standard condition and cultivated to adulthood. For the adult stage, adult worms were collected with basal buffer (0.5 g/L gelatin, 5 mM KPO_4_, 1 mM CaCl_2_ and 1 mM MgSO_4_) and transferred into a 1.5 ml tube. This tube was incubated under heat shock at 33°C for 30 minutes. During this treatment, the tube was inverted every 10 minutes to supply air. The animals were then placed on the plate with food under the standard condition for one hour, followed by the behavioral assays or the analysis of GLR-1::GFP.

### Observation of SNB-1::RFP and GLR-1::GFP

SNB-1 or GLR-1 localization was observed using the Zeiss confocal microscope LSM-510. The animals were immobilized with 1 M NaN_3_. To analyze SNB-1 or GLR-1 localization quantitatively, we measured the intensity and area of puncta of SNB-1 or GLR-1 using ROI. The size of ROI was equivalent in all animals used for these analyses. The average intensity at three random background points was calculated as the background intensity and was subtracted from the intensity of the puncta.

### Observation of the expression of *rig-5ap::venus* at embryonic stage

Adult cuticles were dissolved in lysis solution (NaOH:NaClO = 3:4) to obtain eggs that were then mounted on 5% agar pads with egg salt buffer (5 M NaCl, 2 M KCl). Expression of *rig-5ap*:*:venus* in eggs was observed using the Zeiss confocal microscope LSM-510. *lin-44p::*mRFP expressed in the tail region was used as a transformation marker.

## Additional files

Additional file 1: Figure S1. Proportion of CL phenotype of wild-type, *let-60(lf)* mutants, strains with neuron-specific inhibition, and *let-60*RNAi animals. Animals that exhibited a loopy pattern, both normal and looping patterns (≥30% loopy tracks) and normal patterns (<30% loopy tracks) are defined as strong, mild and normal, respectively. Additional file 2: Table S1. Expression of *let-60* in olfactory neurons and AIY interneurons cannot rescue the CL phenotype. Fraction of animals exhibiting CL in *let-60(lf)* mutants and mutants in which the wild-type *let-60* gene was expressed in AWC olfactory neurons and AIY interneurons. Additional file 3: Figure S2. CL inhibited by chemoattractants. Proportion of animals exhibiting CL in *let-60(n2021lf)* mutants placed in 10^−2^, 10^−4^ or 10^−6^ dilutions of isoamyl alcohol (IAA) (n ≥3 assays). Error bars represent SEM and asterisk significant differences (****P* <0.001, Student’s *t*-test). Additional file 4: Figure S3. Ablation of AWA or ADL neurons did not induce CL. Proportion of wild type animals exhibiting CL with ablation of AWA or ADL neurons by mouse caspase-1 (n ≥3 assays). Error bars represent SEM. Additional file 5: Figure S4. LET-60Ras is not involved in the morphogenesis of IL1, OLQ and RMD neurons. Morphology of IL1 (upper), OLQ (middle) and RMD neurons (bottom) in wild-type animals and *let-60(lf)* mutants. Morphological defects of these neurons are not observed in *let-60(lf)* mutants (yellow arrows, red arrowheads, blue arrowheads and yellow arrowheads = cell bodies, dendrites, axons and neurites, respectively). Scale bar = 10 μm. Additional file 6: Figure S5. SNB-1 localization in IL1 and OLQ neurons is not affected by the *let-60* knockdown. (A, C) Representative images of SNB-1::RFP localization in axons of IL1 in wild type and IL1-specific *let-60* knockdown animals (A) and in axons of OLQ in wild type and OLQ-specific *let-60* knockdown animals (C). Enlarged images demonstrate the axon of IL1 or OLQ neurons (white arrowheads = the clusters of SNB-1::RFP). Scale bar = 10 μm. (B, D) Average intensity and average area of SNB-1::RFP puncta are not affected by cell-specific knockdown of *let-60* in IL1 (B) and OLQ (D) (n ≥8 animals). Error bars represent SEM. N.S. = no significant difference. Additional file 7: Figure S6. Outline of T.C. RNAi. (A) The method of cell-specific RNAi in *C. elegans*. The sense and anti-sense strands of the target gene are expressed by the cell-specific promoter to knock down functions of the gene in the specific cells. (B) We improved the cell-specific RNAi method by adopting the heat shock promoter, which drives the expression in almost all tissues at arbitrary timing by heat shock. Under the standard condition (20°C), expression of only single-stranded RNA is driven by the cell-specific promoter, whereas under the heat shock condition (30°C to 33°C), double-stranded RNA is expressed in only the target cell. The combination of cell-specific promoter and heat shock promoter drives the expression of double-stranded RNA at the optional timing by heat shock. Additional file 8: Figure S7.
*rig-5a* promoter drives expression of Venus from pretzel stage in RMD neurons. (A) Images of eggs at the comma stage (left upper), the plum stage (right upper), the pretzel stage (left bottom) and L1 larva (right bottom). Yellow circle in image of pretzel stage indicates fluorescence of Venus in the head region. From the pretzel stage to L1 larva, expression of Venus was restricted to a low proportion of cells in the head region and was sustained. We confirmed RMD-specific expression of Venus by the *rig-5a* promoter in L1 larva. Red fluorescence represents the expression of *lin-44p*::mRFP in the tail region. Scale bar = 10 μm. (B). Proportion of eggs expressing Venus in the head region at each embryonic stage (n ≥9 animals). Additional file 9: Figure S8. Expression of only one RNA strand does not cause knockdown of target gene. Proportion of animals exhibiting CL in wild type, *let-60(n2021lf)* mutants and animals expressing only one RNA strand (n ≥3 assays). *hsp*::*(s), hsp*::*(as)*, RMD::*(s)* and RMD::*(as)* mean *hsp16-2*::*let-60(s)*, *hsp16-2*::*let-60(as)*, *rig-5ap*::*let-60(s)* and *rig-5ap*::*let-60(as)*, respectively. Error bars represent SEM, and asterisks represent significant differences (***P* <0.01, Dunnett’s test). Additional file 10: Table S2.
*eat-4* mutants exhibiting CL. Proportion of animals exhibiting CL in *let-60(lf)* and *eat-4(ky5)* mutants.
