# Supplementary figures and images for: A role for Ras in inhibiting circular foraging behavior as revealed by a new method for time and cell-specific RNAi
Source: BMC Biol. 2015 Jan 21;13:6. doi: 10.1186/s12915-015-0114-8 (PMC4321700; doi:10.1186/s12915-015-0114-8)

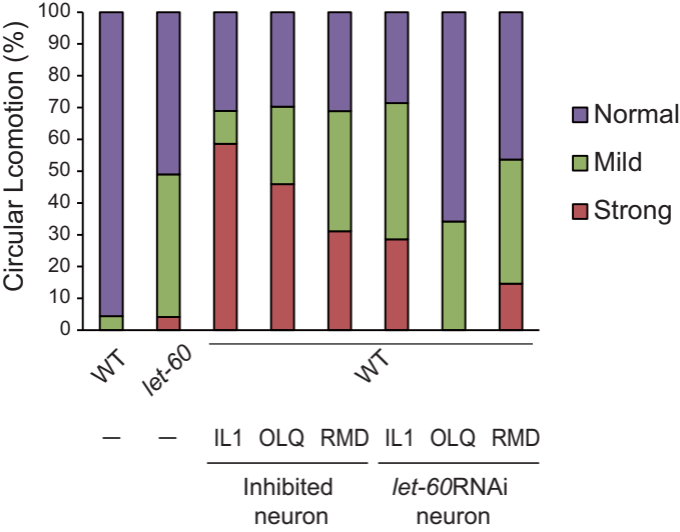

Supplement: Additional file 1: Figure S1. — Proportion of CL phenotype of wild-type, let-60(lf) mutants, strains with neuron-specific inhibition, and let-60RNAi animals. Animals that exhibited a loopy pattern, both normal and looping patterns (≥30% loopy tracks) and normal patterns (<30% loopy tracks) are defined as strong, mild and normal, respectively. [file 12915_2015_114_MOESM1_ESM.pdf]

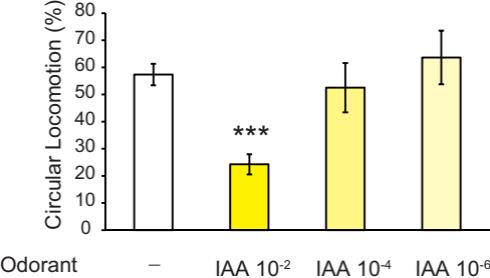

Supplement: Additional file 3: Figure S2. — CL inhibited by chemoattractants. Proportion of animals exhibiting CL in let-60(n2021lf) mutants placed in 10−2, 10−4 or 10−6 dilutions of isoamyl alcohol (IAA) (n ≥3 assays). Error bars represent SEM and asterisk significant differences (***P <0.001, Student’s t-test). [file 12915_2015_114_MOESM3_ESM.pdf]

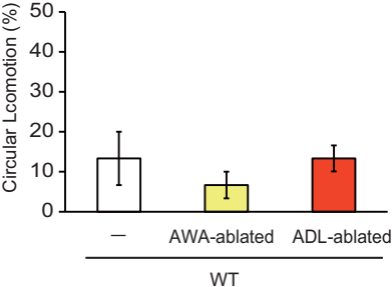

Supplement: Additional file 4: Figure S3. — Ablation of AWA or ADL neurons did not induce CL. Proportion of wild type animals exhibiting CL with ablation of AWA or ADL neurons by mouse caspase-1 (n ≥3 assays). Error bars represent SEM. [file 12915_2015_114_MOESM4_ESM.pdf]

WT

*let-60(lf)* mutants

IL1

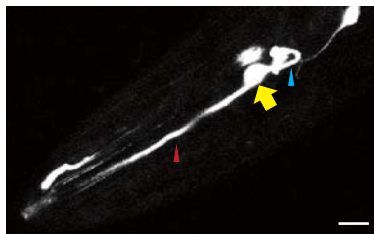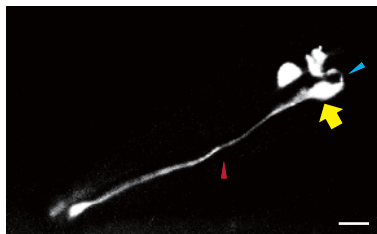

OLQ

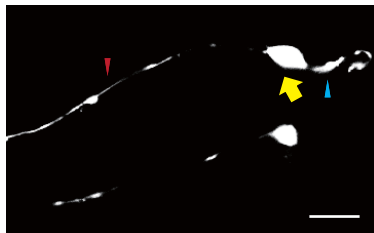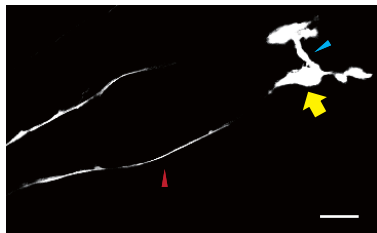

RMD

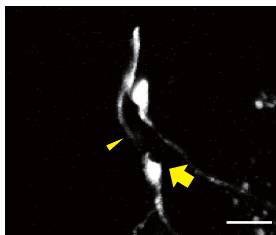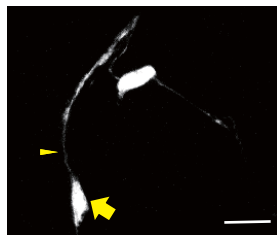

Supplement: Additional file 5: Figure S4. — LET-60Ras is not involved in the morphogenesis of IL1, OLQ and RMD neurons. Morphology of IL1 (upper), OLQ (middle) and RMD neurons (bottom) in wild-type animals and let-60(lf) mutants. Morphological defects of these neurons are not observed in let-60(lf) mutants (yellow arrows, red arrowheads, blue arrowheads and yellow arrowheads = cell bodies, dendrites, axons and neurites, respectively). Scale bar = 10 μm. [file 12915_2015_114_MOESM5_ESM.pdf]

**A**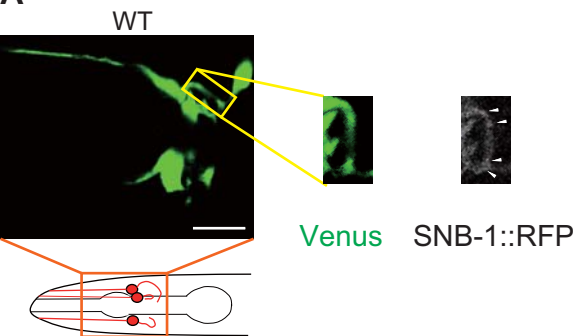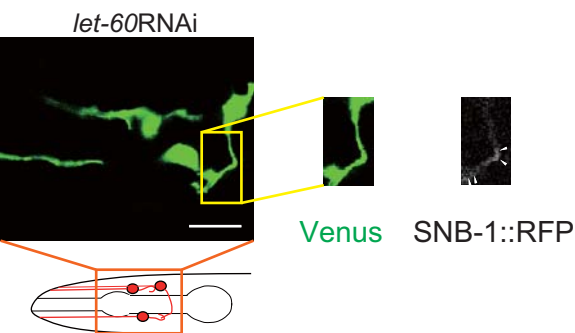**C**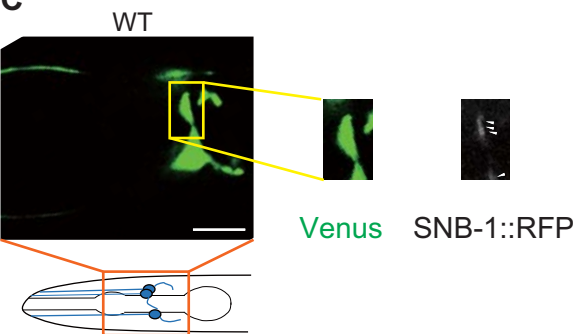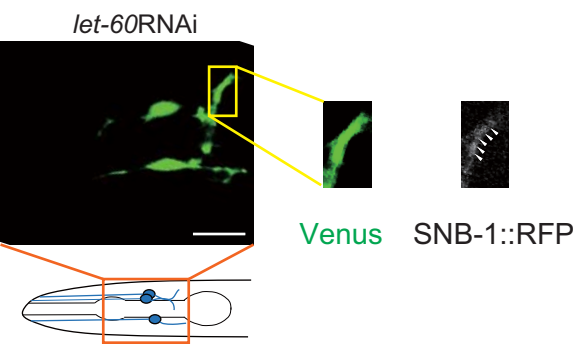**B**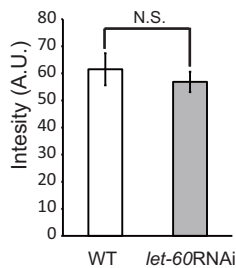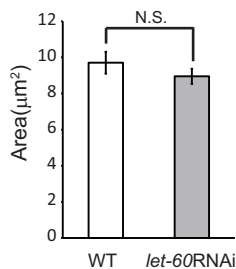**D**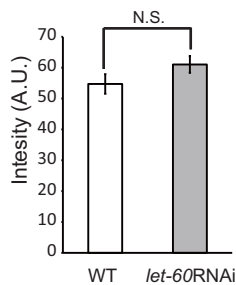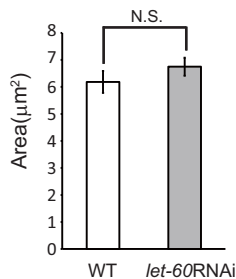

Supplement: Additional file 6: Figure S5. — SNB-1 localization in IL1 and OLQ neurons is not affected by the let-60 knockdown. (A, C) Representative images of SNB-1::RFP localization in axons of IL1 in wild type and IL1-specific let-60 knockdown animals (A) and in axons of OLQ in wild type and OLQ-specific let-60 knockdown animals (C). Enlarged images demonstrate the axon of IL1 or OLQ neurons (white arrowheads = the clusters of SNB-1::RFP). Scale bar = 10 μm. (B, D) Average intensity and average area of SNB-1::RFP puncta are not affected by cell-specific knockdown of let-60 in IL1 (B) and OLQ (D) (n ≥8 animals). Error bars represent SEM. N.S. = no significant difference. [file 12915_2015_114_MOESM6_ESM.pdf]

**A**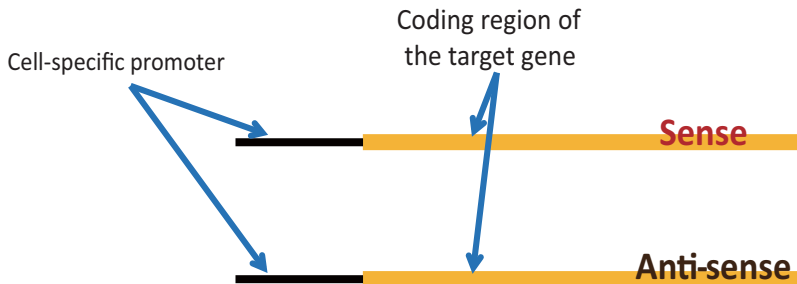**B**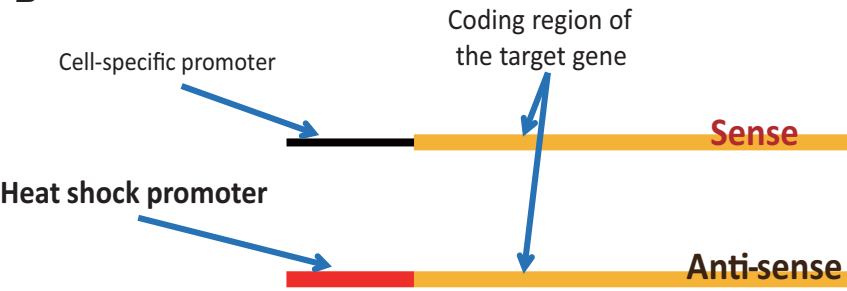

Supplement: Additional file 7: Figure S6. — Outline of T.C. RNAi. (A) The method of cell-specific RNAi in C. elegans. The sense and anti-sense strands of the target gene are expressed by the cell-specific promoter to knock down functions of the gene in the specific cells. (B) We improved the cell-specific RNAi method by adopting the heat shock promoter, which drives the expression in almost all tissues at arbitrary timing by heat shock. Under the standard condition (20°C), expression of only single-stranded RNA is driven by the cell-specific promoter, whereas under the heat shock condition (30°C to 33°C), double-stranded RNA is expressed in only the target cell. The combination of cell-specific promoter and heat shock promoter drives the expression of double-stranded RNA at the optional timing by heat shock. [file 12915_2015_114_MOESM7_ESM.pdf]

A

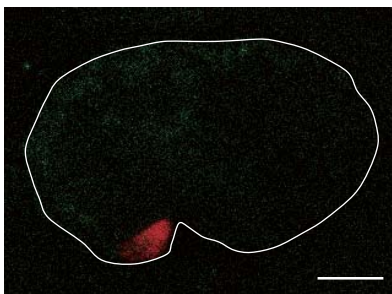

Comma stage

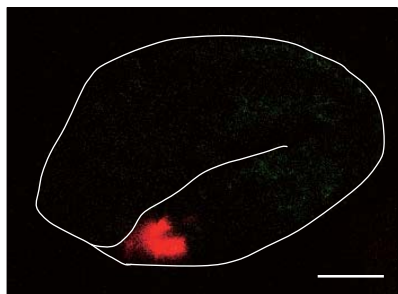

Plum stage

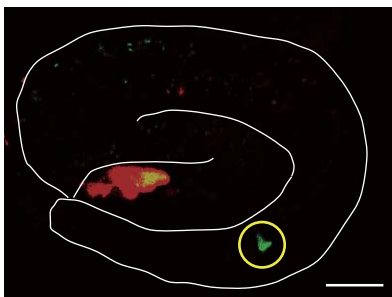

Pretzel stage

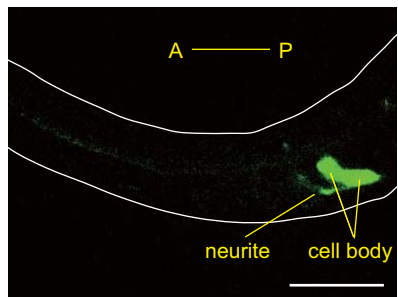

L1 larva

B

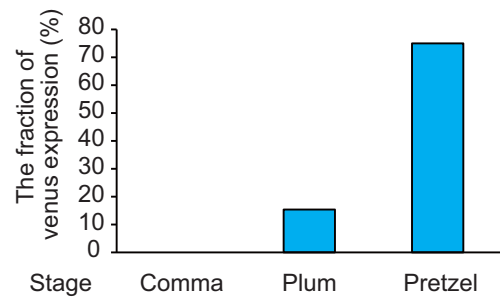

Supplement: Additional file 8: Figure S7. — rig-5a promoter drives expression of Venus from pretzel stage in RMD neurons. (A) Images of eggs at the comma stage (left upper), the plum stage (right upper), the pretzel stage (left bottom) and L1 larva (right bottom). Yellow circle in image of pretzel stage indicates fluorescence of Venus in the head region. From the pretzel stage to L1 larva, expression of Venus was restricted to a low proportion of cells in the head region and was sustained. We confirmed RMD-specific expression of Venus by the rig-5a promoter in L1 larva. Red fluorescence represents the expression of lin-44p::mRFP in the tail region. Scale bar = 10 μm. (B). Proportion of eggs expressing Venus in the head region at each embryonic stage (n ≥9 animals). [file 12915_2015_114_MOESM8_ESM.pdf]

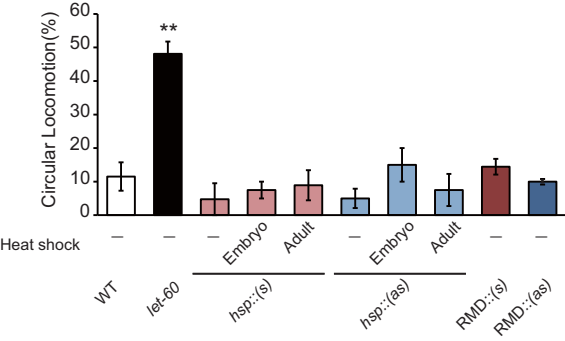

Supplement: Additional file 9: Figure S8. — Expression of only one RNA strand does not cause knockdown of target gene. Proportion of animals exhibiting CL in wild type, let-60(n2021lf) mutants and animals expressing only one RNA strand (n ≥3 assays). hsp::(s), hsp::(as), RMD::(s) and RMD::(as) mean hsp16-2::let-60(s), hsp16-2::let-60(as), rig-5ap::let-60(s) and rig-5ap::let-60(as), respectively. Error bars represent SEM, and asterisks represent significant differences (**P <0.01, Dunnett’s test). [file 12915_2015_114_MOESM9_ESM.pdf]
